# Separation of Soluble Benzene from an Aqueous Solution by Pervaporation Using a Commercial Polydimethylsiloxane Membrane

**DOI:** 10.3390/membranes12111040

**Published:** 2022-10-25

**Authors:** Salam H. Rasheed, Salah S. Ibrahim, Qusay F. Alsalhy, Issam K. Salih

**Affiliations:** 1 Membrane Technology Research Unit, Department of Chemical Engineering, University of Technology-Iraq, Alsinaa Street 52, Baghdad 10066, Iraq; 2 Department of Chemical Engineering and Petroleum Industries, Al-Mustaqbal University College, Babylon 51001, Iraq

**Keywords:** pervaporation, PDMS, benzene, design of experiment, response surface methodology

## Abstract

A developed polydimethylsiloxane (PDMS) membrane was used to separate soluble benzene compounds (C_6_H_6_) from an aqueous solution via a pervaporation (PV) process. This membrane was characterized by scanning electron microscopy (SEM), Fourier-transform infrared (FTIR) spectroscopy, contact angle (CA), and energy-dispersive spectroscopy (EDS). To evaluate the performance of the membrane, the separation factor and permeation flux were estimated in various operating conditions, including the feed temperature, initial benzene concentration, and feed flow rate. The experiments to maximize the separation factor and permeation flux were designed using the response surface method (RSM) that is built into Minitab 18. A quadratic model (nonlinear regression equation) was suggested to obtain mathematical expressions to predict the benzene permeation flux and the separation factor according to the effect of the parameters’ interaction. The optimization of the PV was performed using an RSM that was based on the analysis of variance (ANOVA). The optimal values of the benzene permeation flux and separation factor were 6.7 g/m^2^·h and 39.8, respectively, at the optimal conditions of temperature (30 °C), initial concentration of benzene (1000 ppm), and feed flow rate (3.5 L/min). It was found that the feed concentration was the most influential parameter, leading to a significant increase in the permeation flux and separation factor of the PDMS membrane.

## 1. Introduction

By 2050, the world population is expected to increase by 40–50%, so there is increased interest in providing water suitable for human use [1]. However, preserving the environment and protecting aquatic life from pollution requires disposing of volatile organic compounds (VOCs) using modern, more effective, and less costly methods. Currently, many industrial applications involve the use of organic solvents in their technological processes in order to manufacture refrigerants, plastics, adhesives, paints, petroleum products, etc. [2,3,4,5,6,7]. In the presence of various industrial processes, thousands of cubic meters polluted with these carcinogenic VOC_S_ are thrown into river waters, causing great danger to the environment and humans [8,9,10,11]. Usually, the solubility of VOCs in water is very low, which means that the concentrations of these substances in water are weak and environmentally dangerous. Fortunately, VOCs can be successfully treated using the PV process because it is sufficient for treating these pollutants without the need to use high-cost separation processes, such as distillation, oxidation, biological treatment, and adsorption [12]; these other processes are usually applied, regardless of their high energy demand or their ability to form azeotropes [11,13,14,15,16,17,18]. In contrast, employing the PV process to rid pollutants from industrial water is characterized by being low-cost, having no emission problems, and not requiring expensive regeneration steps [13,14,15,19] as well as using compact/modular designs and allowing for the possible reuse of the recovered VOC solvents [12]. Many researchers have used the PV process for treating water polluted by VOCs under various operating conditions and with different membranes. He et al. used PDMS with lotus leaf powder mixed-matrix membranes (MMMs) and poly(divinylbenzene) (PDVB)-coated PDMS composite membranes to recover ethanol from water [20]. Hamouni et al. prepared PDMS membranes to remove ethanol, toluene, and propanol from water [21], while Unlu used polyvinyl chloride (PVC) to remove propyl acetate [22]. Ethanol was removed using PDMS, PDMS_plasma_C_8_, PDMS–NaCl, PDMS_Al_2_O_3__nat, PDMS_Al_2_O_3__mod._C_8_, PDMS/PVDF (polyvinylidene fluoride), and Pervap™4060 [23]. Peng et al. prepared PDMS membranes by embedded fumed silica nanoparticles that were functionalized with two silane coupling agents—NH_2_(CH_2_)_3_Si(OC_2_H_5_)_3_ (APTS) (3-aminopropyltriethoxysilane) and NH_2_(CH_2_)_2_NH(CH_2_)_3_Si(OC_2_H_5_)_3_ (TSED) (3-triethoxysilylpropyl ethylenediamine)—for the selective separation of ethanol from aqueous solutions [24]. PDMS was employed to remove acetonitrile in experiments by Wang et al. [25], whereas Ye et al. removed phenol by combining PDMS with oleyl alcohol (5%) [26]. Wu et al. prepared PDMS-ZSM-5 zeolite with nylon to remove acetaldehyde [27], while Aliabadi et al. used PDMS to remove styrene [28]. Hilmioglu et al. used PEBA (polyamide and polyether) to remove MTBE (methyl tert-butyl ether) [29]. Khayet et al. used PDMS Pervap™4060 to remove acetone [30]

Of the VOCs, benzene (C_6_H_6_, the pollutant under study) is a volatile substance with a hexagonal cyclic molecule; it is a flammable liquid that is nonpolar, colorless, and has great thermal stability. Benzene is used to prepare many chemicals, such as phenol, styrene, cyclohexane, polyester resins, aniline, chlorobenzenes, and alkylbenzenes, and is used in the production of drugs, dyes, insecticides, and plastics [16]. Benzene is also produced from refinery operations [17]. The presence of benzene as a pollutant in drain water is considered very dangerous for humans and the environment, as benzene causes many carcinogenic diseases [18]. Therefore, water polluted by benzene must be treated before being discharged into rivers or other bodies of water. Uragami et al. [31] used the PV process to separate benzene from an aqueous solution using two different membranes. The first was polyvinyl chloride (PVC) with a 13.8 cm^2^ active area under the following operating conditions: 500 ppm initial concentration of benzene in the feed, 40 °C feed temperature, and a vacuum pressure of 1.33 Pa abs [32]. The normalized permeation flux was 1.39 × 10^−5^ kg m/m^2^·h, and 8.1 wt% of benzene reached the permeate. The second was polyvinyl chloride with 1-allyl-3-butylimidazilium bis(trifluoromethanesulfonyl)imide ([ABIM]TFSI), where [ABIM] TFSI, as an ionic liquid, has a high affinity for VOCs and a low affinity for water. Under the same conditions, the normalized permeation rate was 1.91 × 10^−5^ kg m/m^2^·h, and 38.4 wt% of benzene reached the permeate.

Ohshima et al. [32] fabricated organic–inorganic membranes by using the sol–gel process using poly(methyl methacrylate-co-vinyltriethoxysilane) (P(MMA-co-VTES)) and poly(butyl methacrylate-co-vinyltriethoxysilane) (P(BMAco-VTES)) as organic compounds with good affinities for VOCs and tetraethoxysilane (TEOS) as an inorganic compound to separate benzene from an aqueous solution. The PV process was achieved at a temperature of 40 °C, a vacuum pressure of 0.01 mm Hg abs, and an initial benzene concentration in the feed of 500 ppm. The experimental results indicated that the benzene–water selectivity of the P(BMA-co-VTES)/TEOS hybrid membrane was about 20 times higher than that of the P(MMA-co-VTES)/TEOS hybrid membrane. Peng et al. [33] added a carbon molecular sieve (CMS) to the PDMS membranes as a filling to remove the benzene from the aqueous solution by the PV process. The membrane’s active area and pressure vacuum were 28 cm^2^ and 1.0 kPa abs, respectively. The separation factor and flux were evaluated under several operating conditions by varying the initial concentration of benzene, the feed temperature, the feed Reynold numbers, and the effects of the CMS content in the PDMS.

The performances of PDMS membranes vary according to the main components of their compositions, which mainly consist of various proportions of PDMS polymer types (different molecular weights), solvent types, crosslinking types, and catalyst types as well as various materials that may be added to improve the membrane performance such as nanomaterials, carbon molecular sieves, etc. The thickness of the effective layer of the PDMS membrane also has a significant effect on the membrane performance. The mechanical properties of the PDMS membrane have been improved by using many types and several numbers of supporting layers. Thus, the performance of PV processes for VOC recovery from aqueous solutions in terms of the permeation flux and separation factor were mainly based on the type of PDMS membrane, the operating conditions, and the operation style (batch or continuous), as shown in Table 1. It is clear that there are differences in PV process performances. The membrane performance plays a significant role in the PV process results, as shown in Table 1, and the operation style and operating conditions play an important role in the performance results. The batch style of low feed volume leads to a continuous change in the VOC concentration in the feed during operation, which leads to a drop in the VOC permeate flux across the membrane, where the concentration of the VOCs in the feed is considered the highly affected operator for the performance of the PV process, as shown in the present results.

Many researchers used RSM for experimental design and applied a statistical analysis in their work, such as Wee Shin Ling et al. [38], who applied the design of experiments coupled with RSM to study the dehydration of an isopropanol–water mixture by the PV process, where a commercially available ceramic membrane was used to achieve this process. The results showed the effects of feed temperature, feed concentration, vacuum pressure, and feed velocity on the membrane performance. Moreover, the optimal operating conditions and a quadratic model were obtained.

Khayet [39] et al. used a commercial PDMS membrane to remove acetonitrile from the aqueous solution by the PV process under different conditions of temperature and initial organic concentration in the feed. The overall mass transfer coefficients and the activation energy associated with the permeation process were determined. A statistical experimental design and RSM were used to obtain the optimal conditions of the pervaporation process, where the permeate flux ratio and the concentration of organic in the permeate represented the responses.

Margarida Catarino et al. [40] used an olyoctylmethylsiloxane/polyetherimide (POMS/PEI) composite asymmetric membrane to separate aroma from beer by the PV process at various operating conditions of the feed temperature, feed velocity, and permeate pressure. The RSM method has been applied to show the effects of the factors mentioned above on the responses that were represented by the permeate flux, the aromas/ethanol selectivity, the ethanol concentration, and the ratio between high alcohol and ester concentrations on the permeate by building a mathematical model; moreover, the optimal conditions were determined.

The mechanism of solution diffusion is one of the most widely accepted models for describing the movement of matter through a dense membrane in the PV process [41]. The membranes used in PV processes are nonporous and often depend on the solution diffusion mechanism, which consists of three steps. The first one is sorption, where the target substance is sorbed on the membrane surface. The second step is diffusion through the membrane, and this step depends on the affinity between the material and the membrane, where molecules penetrate through polymeric chains. The third step is desorption, where the phase of the material changes from liquid to vapor due downstream vacuum pressure [42].

A commercially developed polydimethylsiloxane (PDMS) membrane prepared by DeltaMem AG (Switzerland) for the pervaporation process was used to separate various VOCs from aqueous solutions, such as ethanol [23], acetone and acetonitrile [30], and butanol [43]. However, to date, no work has been presented in the literature using this PDMS membrane to separate soluble benzene from an aqueous solution. Therefore, the current study is focused on testing this membrane’s performance using the PV process for the separation of the soluble benzene compound from an aqueous solution at different operating conditions, including the feed temperature, the initial concentration of benzene in the feed, and the feed flow rate. RSM was used in this study to obtain the best operating conditions to obtain an optimal response during the PV process and to determine which of the operating parameters have the most significant effects on this response. In addition, this study created a mathematical expression estimated by a data regression that linked the significant variables with the predicted response.

## 2. Materials and Methods

### 2.1. Materials

The commercial hydrophobic membrane (PDMS Pervap^TM^4060) used in this research was supplied by DeltaMem AG, Allschwil, Switzerland. The benzene (99.5% purity) was provided by Riedel-De Haën AG Seelze-Hannover, Wunstorfer, Germany. Distilled water was used to prepare all aqueous solutions.

### 2.2. Membrane Characterizations

Many membrane characterization tests were conducted to identify the characteristics of the commercial membrane, including the following:

#### 2.2.1. Contact Angle

It is well-known that the contact angle (CA) is indicated by the hydrophobicity or hydrophilicity of the membrane. Thus, the contact angle of the present PDMS membrane used was measured using CAM 110-04W, Taiwan. The contact angle was measured by putting a drop of distilled water on the active layer of the membrane, where the contact angle between the drop and the surface of the membrane was read.

#### 2.2.2. Scanning Electron Microscopy (SEM)

Scanning electron microscopy is the most common technique for morphological membrane characterization. For enclosing the membrane morphology, the cross section of the membrane was viewed by scanning electronic microscopy (SEM) by the TESCAN VEGA3 SB Instrument EO-Service, Kohoutovice, Czech Republic. By using liquid nitrogen, the membrane was cut to obtain a clean fracture and a clear image.

#### 2.2.3. Fourier-Transform Infrared Spectroscopy (FTIR)

FTIR is a technique that is used to obtain the infrared spectrum of absorption, emission, and photoconductivity of solids, liquids, and gases. It is used to detect different functional groups. The functions groups in the PDMS membrane were determined by using a Tensor 27 FTIR spectrometer, from Bruker, Ettlingen, Germany. 

#### 2.2.4. Energy-Dispersive Spectroscopy (EDS)

EDS is an analysis method that identifies the elemental and chemical compositions of a substance. This test was carried out on the membrane in the present study by the TESCAN VEGA3 SB Instrument EO-Service, Kohoutovice, Czech Republic.

### 2.3. Pervaporation Process

**Figure 1:**
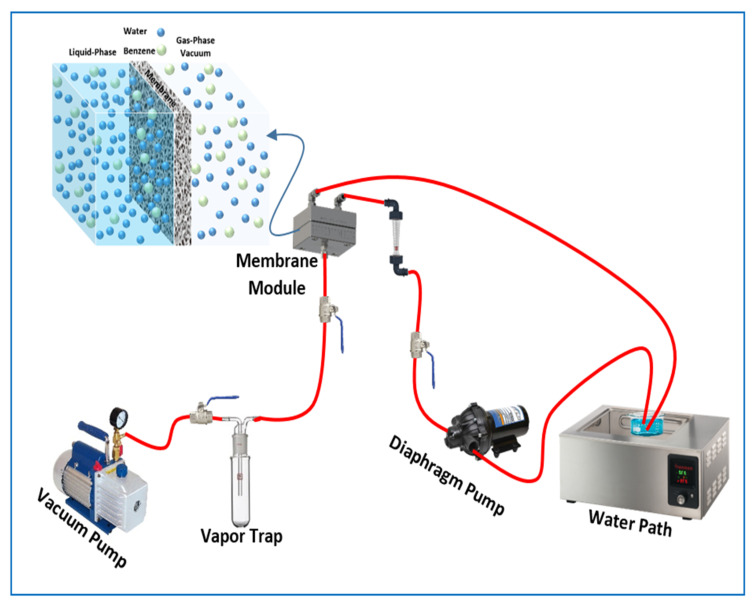
Schematic diagram of the pervaporation process.

Pervaporation process tests were carried out using a lab-scale apparatus, which is illustrated schematically in Figure 1. First, a 200 mL benzene–water mixture was prepared in a 250 mL glass flask. To control the temperature, the flask was placed in a thermal digital water bath (DK-8AXX, MEDITECH, Taichung, China), with the bath set at temperatures ranging from 30 to 50 °C. The mixture feed was pumped to the membrane cell by a diaphragm pump (BD, 400GPD, Taiwan), where the initial concentration of the benzene in the feed solution ranged from 100 to 1000 ppm and the feed flow rate ranged from 1.5 to 3.5 L/min. This membrane, with an effective area of 48 cm², rested on a perforated plate placed in the middle of the PV cell to support the membrane. Downstream of the module, the vacuum pressure was kept at 2.0 kPa by a single-stage vacuum pump (B-42, Sigma, Shanghai, China). Samples of the permeate were collected in a vapor trap that was immersed in liquid nitrogen. The collected permeate was weighed with a digital balance with a precision of 0.001 g. The concentration of benzene and water in the permeate was estimated by measuring at least in triplicate using an ultraviolet-visible spectrophotometer (V-630, Jasco, λmax = 253 nm), Tokyo, Japan. 

The membrane performance in the PV process could be evaluated in terms of the permeation flux (*J*) and separation factor (*S.F.*). The permeation flux is the rate of transport of the targeted substance through a unit area of a membrane during a given time; the separation factor consists of two materials (*i* and *j*), such as benzene-water, and it is defined as the ratio of the mole fraction of the components in the permeate to that in the feed.

The flux and separation factor were determined according to Equations (1) and (2):

(1) $$J=\frac{w}{A\;t}$$

(2) $$S.F.=\frac{y_{i}/yj}{x_{i}/x_{j}}$$

where w is the weight of the permeate; t is the experimental time; A is the effective area of the membrane; and $y_{i}$, $y_{j}$, $x_{i}$, and $x_{j}$ are the mole fractions in the permeate (y) and the feed (x) in relation to benzene and water, respectively.

### 2.4. Experimental Design

While other studies have employed a variety of methods to design similar experiments (e.g., the Taguchi method) [44,45], this paper is based on the response surface method (RSM), which was designed using Minitab 18. RSM is an assortment of mathematical and statistical techniques to create, improve, and optimize processes and can be utilized to assess the relative significance of several factors, even in the presence of complex interactions [46]. Therefore, in this work, the system required 20 experimental runs in which all factors were varied simultaneously over a set of experiments to determine the relationships between the factors affecting the output. The experimental design program was also used to obtain the best operating conditions and to determine the optimal response [39,47].

## 3. Results and Discussion

### 3.1. Membrane Characterizations

#### 3.1.1. Contact Angle

**Figure 2:**
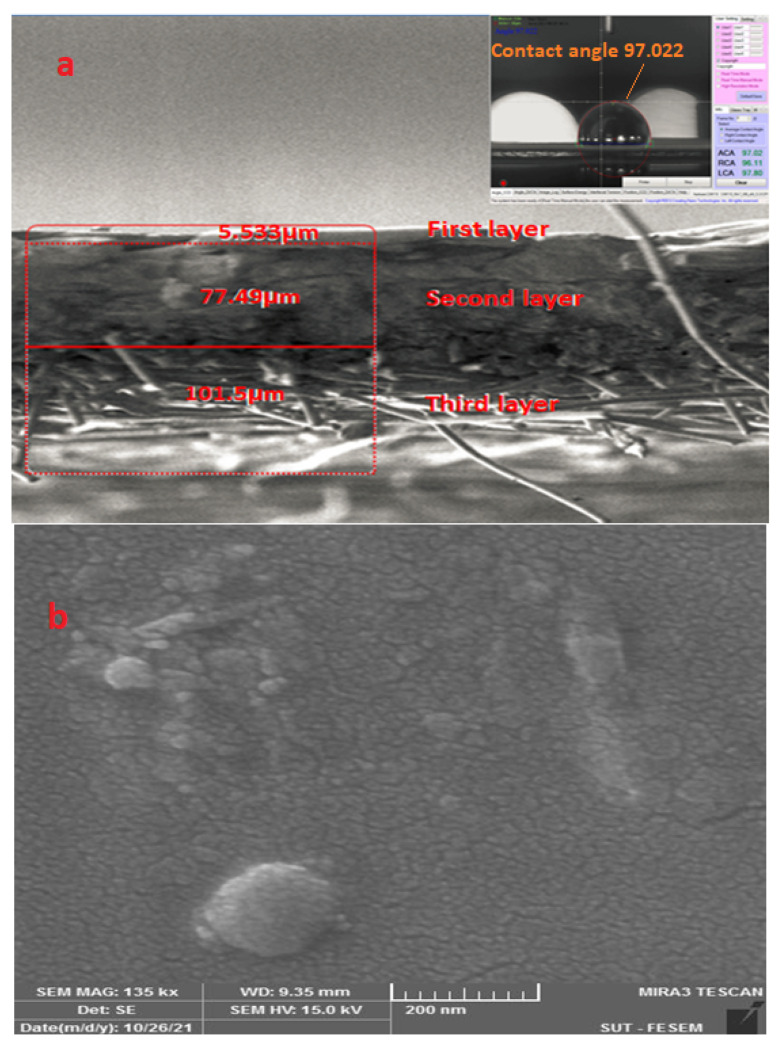
(**a**) Contact angle of PDMS membrane and SEM image of PDMS membrane cross section (200 µm). (**b**) SEM image of PDMS membrane surface.

Because the contact angle (CA) indicates the hydrophobicity or hydrophilicity of a membrane, the contact angle of the PDMS membrane in this study was measured. Measurements were repeated three times, and all results were >90°, as can be seen in Figure 2a. The average CA result was 97° ± 0.05, demonstrating that the membrane was hydrophobic.

#### 3.1.2. SEM Analysis

To examine the membrane’s morphology, its cross section was viewed by scanning electron microscopy (SEM). The SEM tests of the PDMS membrane are presented in Figure 1a, which clearly shows that the membrane contains three layers: (1) an active layer of 5.53 µm thickness, characterized as a dense (nonporous) layer; (2) a porous layer of 77.49 µm thickness; and (3) a nonwavy fabric layer with a thickness of 101.5 µm. The membrane surface of the first layer was smooth and uniform, as shown in Figure 2b. The dense nature of the membrane is confirmed by the image in this figure.

#### 3.1.3. FTIR

**Figure 3:**
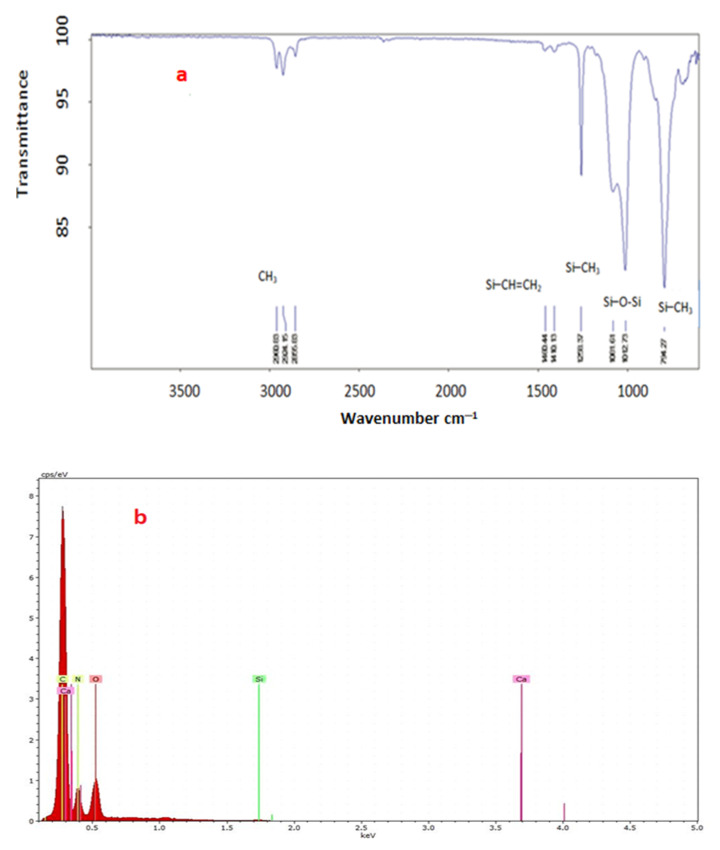
(**a**) FTIR spectra of PDMS membranes. (**b**) EDS analysis of PDMS membranes.

The functional groups in the PDMS membrane were determined, and the surface composition of the PDMS membrane was analyzed, as illustrated in Figure 3a. The peak at 794 represented a Si–CH_3_ group, and the peaks from 1012 to 1061 pointed to a Si–O–Si group. The peak at 1258 represented a Si–CH_3_ group, while the peak at 1460 represented a Si–CH=CH_2_ group. The peaks at 2856 and 2960, respectively, corresponded with the asymmetric and symmetric vibration of CH_3_.

#### 3.1.4. Energy-Dispersive Spectroscopy (EDS)

EDS is an analysis method that identifies the elemental and chemical compositions of a substance. Figure 3b presents the results of an EDS test to identify the elements of the PDMS membrane.

It is noted that the presence of silicon, carbon, nitrogen, and oxygen, in addition to calcium, which may appear as a result of the use of some additives by the manufacturing company, may be to improve the performance of the membrane.

### 3.2. Influence of Feed Temperature in Benzene–Water Mixture

**Figure 4:**
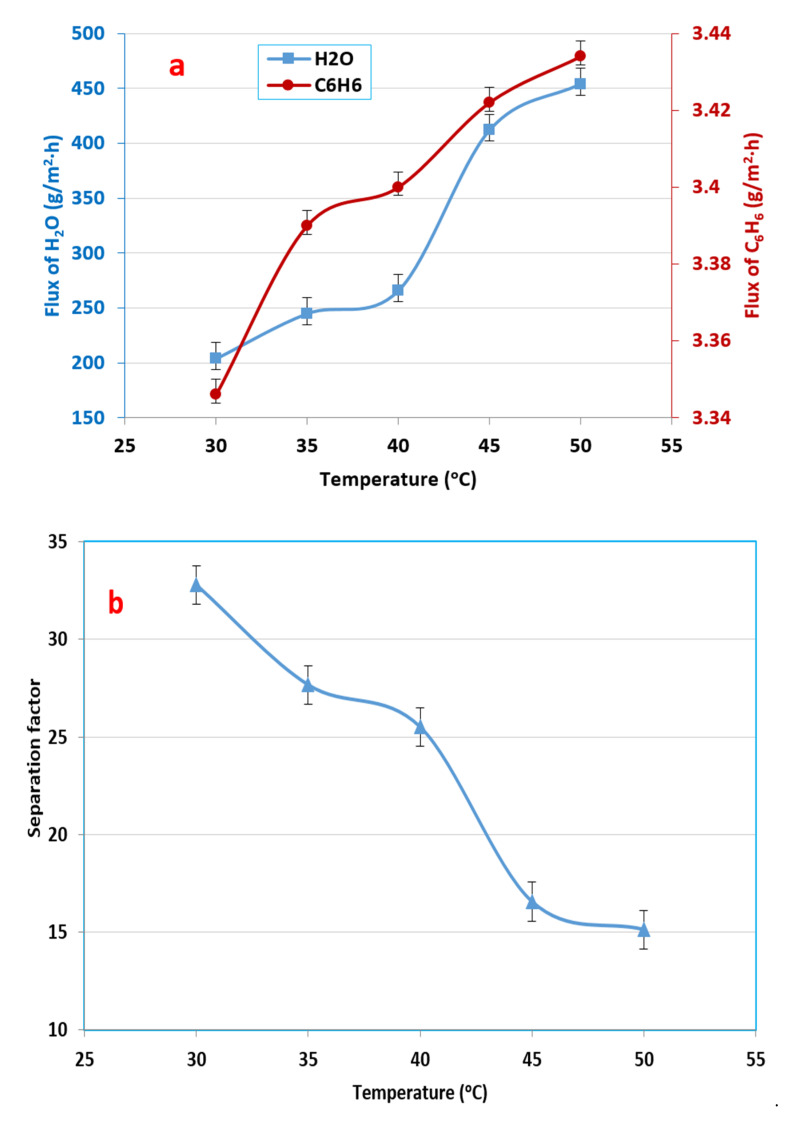
Effect of temperature on (**a**) the fluxes of benzene and water and (**b**) the separation factor, at an initial benzene feed concentration of 500 ppm and a 3 L/min feed flow rate.

The experimental results of the influence of temperature on the permeation flux are shown in Figure 4a, which demonstrates that the benzene permeate flux through the PDMS membrane increased from 3340 to 3434 mg/m^2^·h, while the water flux increased from 204 to 454 g/m^2^·h. This figure clearly shows that the permeate flux increased with a rise in temperature due to the increment in the distance between the polymer chains with the rise in temperature; this resulted in an increase in the free volume available for molecular transit [48]. Moreover, by increasing the temperature, the vapor pressure of each compound increased, which led to a high permeation flux of all compounds by increasing the driving force across the membrane [28].

However, in spite of the permeation flux increasing, the separation factor decreased with a rise in temperature, as displayed in Figure 4b. The fluxes of benzene and water increased with an increase in temperature, but the rate of increase of the water molecules was greater than that of the benzene molecules. The separation factor changed from 32.78 to 15.12, possibly due to benzene having a larger molecular size than water. Therefore, with the increase in the feed temperature, the rate of water diffusion should increase faster than benzene [34,41].

### 3.3. Influence of the Initial Feed Concentration in Benzene–Water Solution

The solubility of the benzene component in water can reach 1800 ppm at 25 °C [49]; consequently, the initial concentrations of benzene studied in the present work ranged from 100 to 1000 ppm. The experimental results displayed that the increase in the feed concentration led to an increase in the benzene flux.

**Figure 5:**
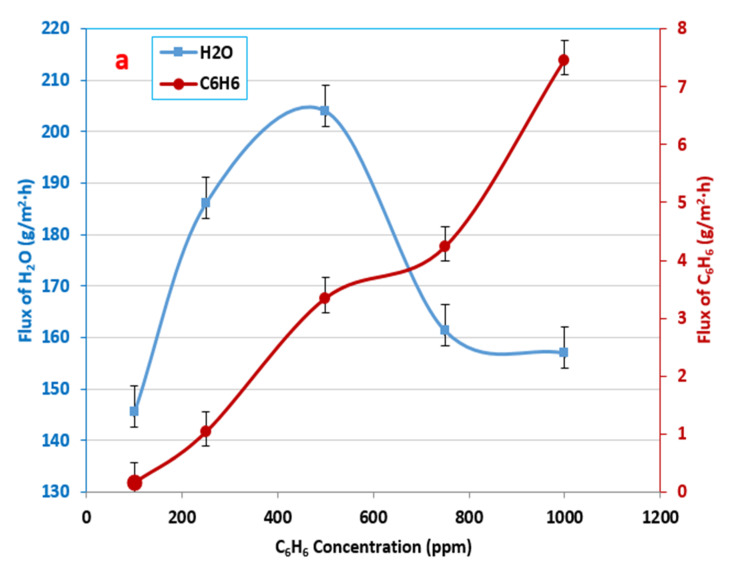

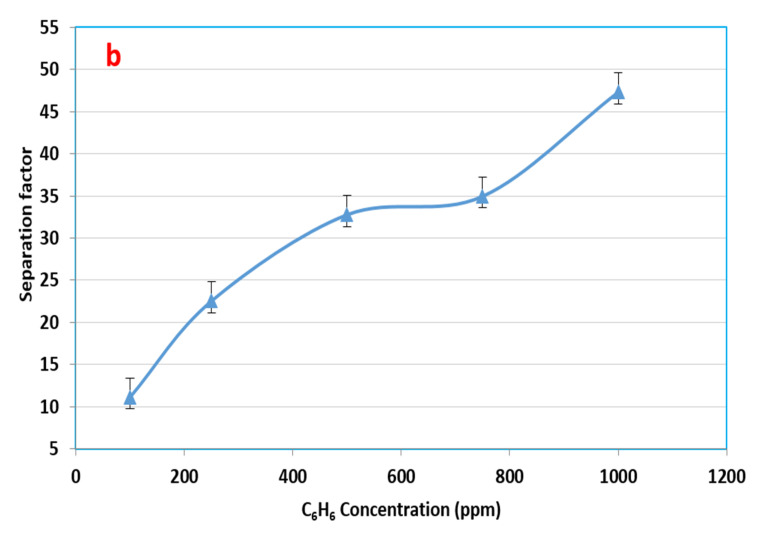
Effect of the initial benzene feed concentration on (**a**) the flux of benzene and water and (**b**) the separation factor at a feed temperature of 30 °C and a 3 L/min feed flow rate.

With an increase in the initial benzene concentration in the feed solution from 100 to 1000 ppm, the permeation flux increased from 0.161 to 7.45 g/m^2^·h, as shown in Figure 5a. The increase in the initial benzene concentration in the feed caused an increase in the driving force between the upstream and downstream pressures across the membrane [34].

The water flux behavior changed with an increased initial benzene concentration in the feed solution. At first, the water flux increased and achieved 204 g/m^2^·h. Then, it decreased to 157 g/m^2^·h, as can be seen in Figure 5a. This result may be explained by the fact that the water molecules clustered due to the hydrogen bonding between the water molecules, which reduced their diffusivity and permeability [25]. The separation factor also increased with an increase in the initial benzene concentration in the feed solution, as shown in Figure 5b, where water clustering developed in the membrane, arising from the repulsive interaction between the organic compound and water that had been absorbed [48]. It was proven that the water permeation within polymer membranes may be hindered by the formation of a water cluster. This analysis revealed that because the diffusion size of the water increased, the diffusion coefficient decreased [28].

### 3.4. Effect of Feed Flow Rate on the Benzene Flux

**Figure 6:**
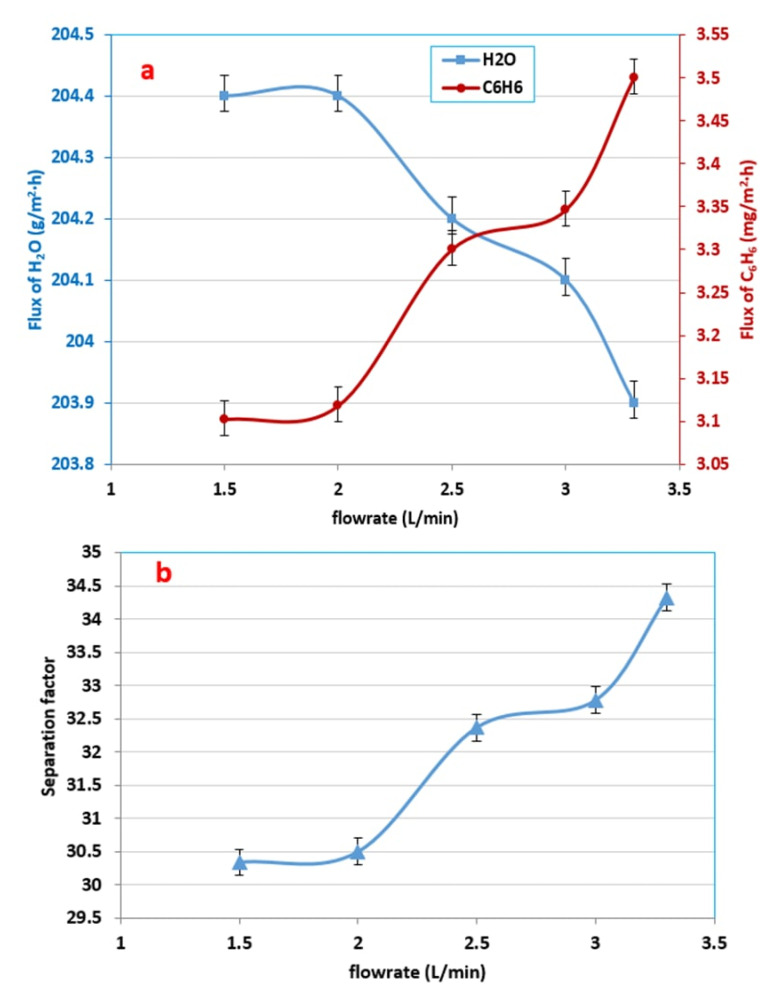
Effect the feed flow rate on (**a**) the fluxes of benzene and water and (**b**) the separation factor at a 500 ppm initial benzene feed concentration and a 30 °C feed temperature.

Figure 6a demonstrates that there was an improvement in the benzene partial flux with increases in the feed flow rate at a 500 ppm initial concentration of benzene and a temperature of 30 °C. The experimental results showed that when the flow rate increased from 1.5 to 3.5 L/min the permeate flux of benzene increased from 3102 to 3500 mg/m^2^·h. The effect of concentration polarization may explain these results because it is well-known that this effect occurs at the liquid boundary layer near the surface of the membrane. Concentration polarization always decreases the rate of permeation of the more permeable compound (benzene) and increases the rate of permeation of the less permeable compound, such as water; that, in return, decreases the efficiency of the separation. However, the increase in the flow rate of the feed could decrease the influence of concentration polarization, and the boundary layer thickness would also decrease. Thus, the resistance to the transfer of the material through the membrane decreased for benzene, while the flux of water seemed to be slightly decreased from 204.4 to 203.9 g/m^2^·h, which was almost constant (see Figure 6a). In fact, the permeate flux of water is mainly depends on the rate of diffusion across the membrane, which means that should be independent of feed flow rate [29,48].

The separation factor increased with increasing feed flow rates, as depicted in Figure 6b, where the flux of benzene increased while the water flux decreased very slightly (approximately constant) [33]. While increasing the flow rate of the feed, an increase in the flux of benzene and a slight decrease in the flux of water was observed. Therefore an increase in the separation factor was obtained according to Equation (3) [50]:

(3) S.F.=ciperm.cj perm.ci feedcjfeed=JiJjci feedcjfeed

where $c_{i}perm.$ and $c_{i\;}feed$ are the concentration of compound i in the permeate and feed, respectively; $c_{j\;}perm.$ and $c_{j\;}feed$ are the concentration of compound *j* in the permeate and feed, respectively; and $J_{i}$ and $J_{j}$ are the permeate flux of compounds *i* and *j*, respectively.

The performance of the present PV process for soluble benzene recovery from an aqueous solution is mainly based on the commercial PDMS membrane used in the present work, the operating conditions, and the operating style. This performance may be different than the performances reported in previous works according to the characterizations of the membrane used, the operating conditions, and the style. Table 1 shows the performances of many PV processes with different VOC contaminants in aqueous solution using many types of PDMS membrane. As mentioned above, this table shows various performances in terms of permeation flux and separation factor according to the operators listed in Table 1. A comparison of the performance between the present and previous works of the PV process for benzene recovery from an aqueous solution is shown in Table 2. The performance of the present study seems to be lower in comparison with the performances that were reported in some previous studies. The expected reason may be due to the differences in the performance of the membrane used, the operating conditions, and the operating style. In the present work, the operation was based on a certain initial benzene concentration in a relatively small feed mixture volume (200 mL) that led to a significant decrease in the benzene concentration in the feed during the experiment’s operation. This operation style is somewhat similar to the operation style used by Xianshe Feng and Robert Y.M Huang [51] for isopropanol recovery from an aqueous solution, where they also used a small volume of an isopropanol–water mixture near to that used in the present study but with a high initial concentration of isopropanol.

### 3.5. Results of the RSM

#### 3.5.1. Predicted Model and ANOVA Calculations

The operating parameters of the RSM experimental data points were obtained using Minitab 18, and the experimental results that represented the responses of the process (i.e., the permeate flux and separation factor) are provided in Table 3.

The analysis of these results was used to determine mathematical expressions to predict the responses of the PV process (i.e., the benzene permeate flux and separation factor). A quadratic nonlinear regression model was suggested using Minitab 18 to obtain the equations for the benzene permeate flux and separation factor, as follows:

(4) $$J_{B}=A_{0}+A_{1}\;T+A_{2}\;C-A_{3}\;F+{\;A}_{4}{\;T}^{2}+A_{5}C^{2}+A_{6}{\;F}^{2}+A_{7}\;T\;C+{\;A}_{8}T\;F\;+{\;A}_{9}\;C\;F$$

(5) $$S.F.=A_{0}+A_{1}\;T+A_{2}\;C+A_{3}\;F+{\;A}_{4}{\;T}^{2}+A_{5}C^{2}+A_{6}{\;F}^{2}+A_{7}\;T\;C+{\;A}_{8}T\;F\;+{\;A}_{9}\;C\;F$$

where the coefficients from A_0_ to A_9_ are illustrated in Table 4.

**Figure 7:**
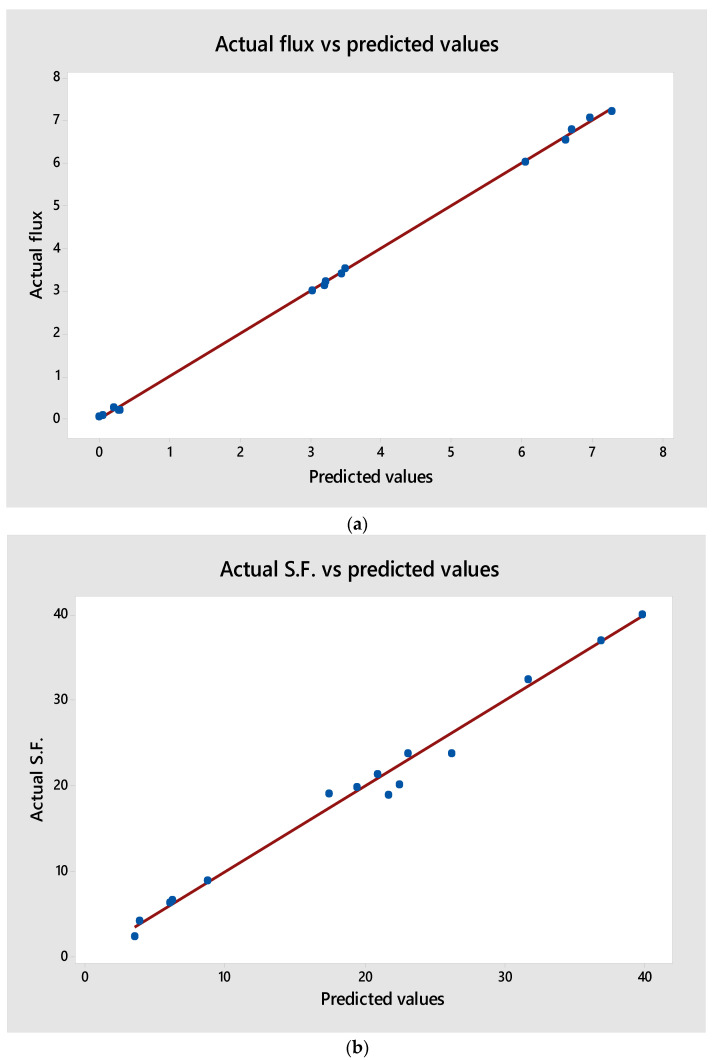
The relationship between the actual and predicted (**a**) benzene flux and (**b**) S.F.

The predicted values for the benzene permeate flux and separation factor from Equations (4) and (5) were compared with the experimental values from the experiments given in Table 2, as presented in Figure 7a,b. This figure indicates that the proposed models (regression formulas) were in good agreement with the experimental results.

To fully quantify the significance of each factor, an analysis of variance (ANOVA) was calculated using Minitab 18. Table 5 and Table 6 display the results of the ANOVA calculations for the benzene permeate flux and separation factor, respectively. On the other hand, the correlation coefficients (R^2^ values) were found to equal 99.95 and 98.63 for the benzene permeation flux and separation factor, respectively, both of which are considered desirable. This reveals that about 99% of the data deviation can be described by the two empirical models [39].

Moreover, the predicted correlation coefficient (R^2^pred) values are close to the adjusted correlation coefficient (R^2^adj) for both models, as shown in Table 7. This means that significant terms were included in both empirical models.

#### 3.5.2. Optimization of PV Process

**Figure 8:**
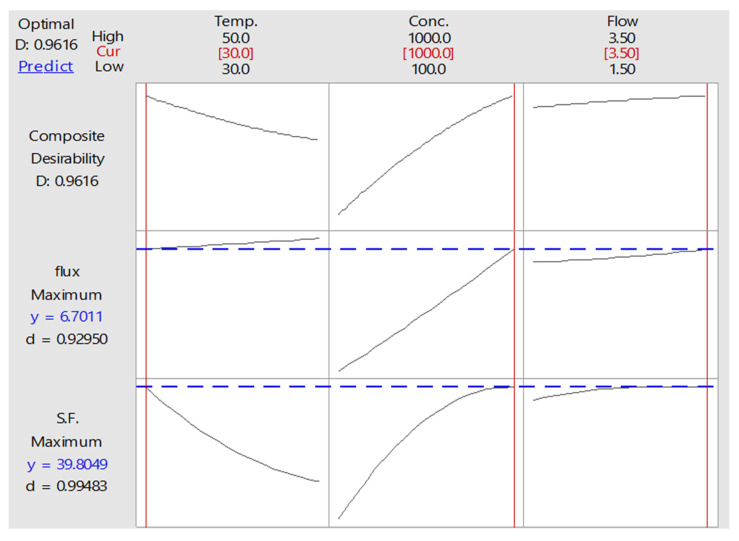
Optimization plot for benzene flux and S.F. for benzene–water solution.

In the optimization of multiple response processes in the diverse field of applied science and engineering, the desirability function approach is one of the most widely used methods. This method combines the individual desirability of multiple responses into a single value ranging from 1.0 to 0. A value of 1 is the ideal case, so values nearer to 1 are the most desirable in terms of describing the optimal operating conditions. However, when this value is close to zero, it indicates that one or more responses fall outside the desirable limits [57,58,59,60,61,62,63]. Thus, by using Minitab 18, the calculation of the desirability function of the two present responses (the benzene flux and S.F) combined the individual desirability into a single number, as given in Table 8. This table identifies the best operating conditions studied (i.e., the temperature, concentration, and flow rate) because these three factors were estimated to have the greatest influence on maximizing the benzene permeation flux and separation factor. As such, Figure 8 and Table 8 show the results of the desirability function for the separation of benzene from the aqueous solution.

#### 3.5.3. Response Surface Plots of Multiple Effects

**Figure 9:**
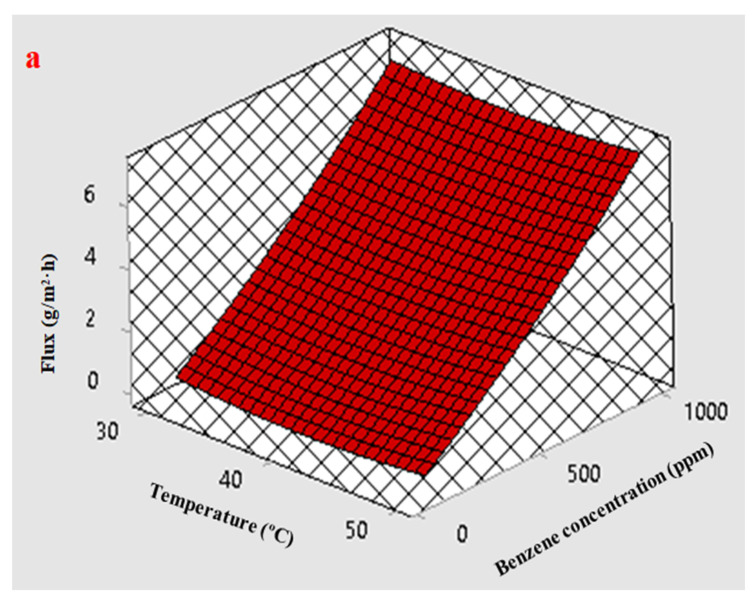

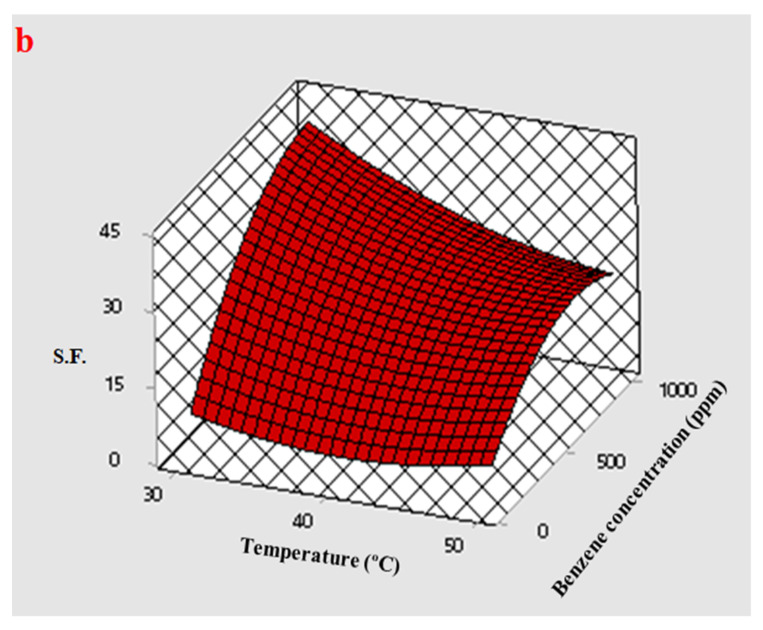
Effect of the feed temperature and the benzene concentration on (**a**) benzene flux and (**b**) separation factor (S.F.) at a feed flow rate 2.5 L/min.

Figure 9a presents the response surface plot that illustrates the effect of the initial benzene feed concentration and temperature on the benzene permeate flux. This figure shows that with increases in both the temperature of the feed and the initial benzene concentration the benzene permeation flux also increased. A rise in the temperature of the feed from 30 to 50 °C led to a slight improvement in the benzene flux, while an increase in the initial benzene concentration was more effective than the temperature of the feed and caused a clear increase in the benzene permeation flux.

The response surface plot in Figure 9b illustrates the effect of the initial benzene concentration in the feed as well as the effect of the feed temperature on the separation factor. Increasing the temperature led to an increase in the possibility of swelling of the membrane, which had a negative effect on the S.F. Thus, due to the swelling of the membrane when the temperature rose, the S.F. decreased, which means that the amount of water permeation through the membrane was greater than the benzene permeation; in contrast, an increase in the initial benzene concentration caused an increase in the S.F. The enhancement of the S.F. may be explained by the fact that the water molecules clustered due to the hydrogen bonding between the water molecules, which reduced their diffusivity and permeability. However, due to the interaction between the factors, an increase in the initial concentration of benzene in the feed improved the S.F. in spite of the temperature being higher.

**Figure 10:**
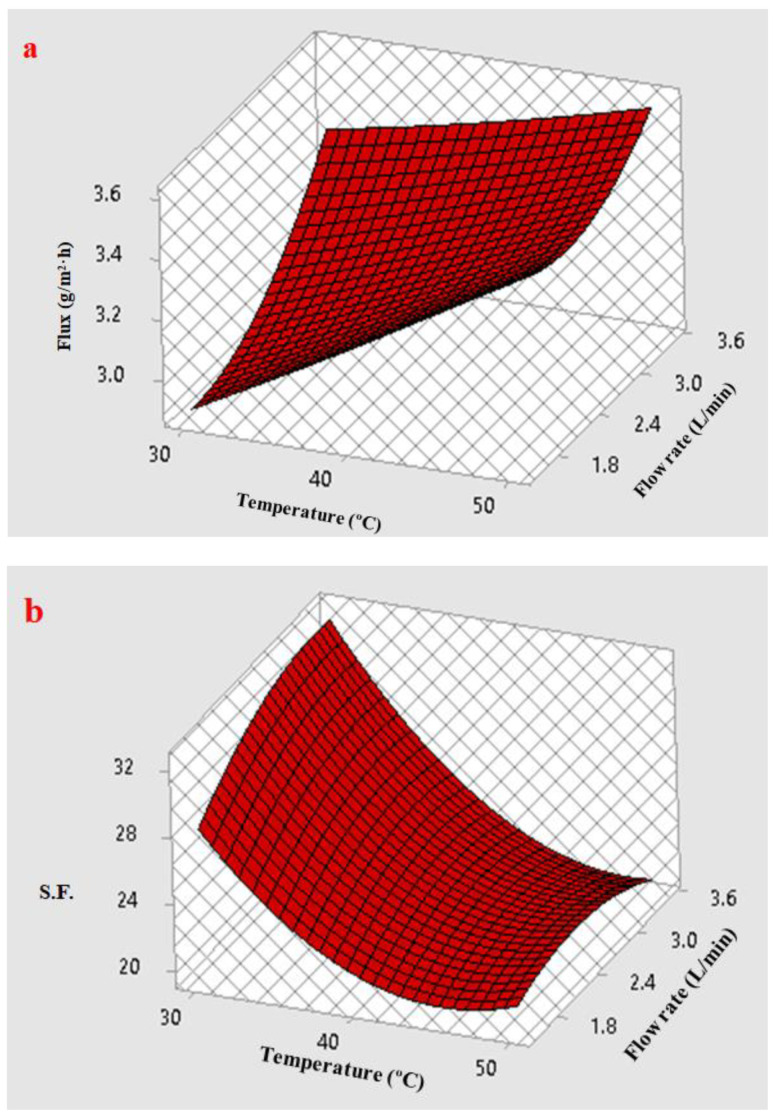
Effect of the feed temperature and feed flow on (**a**) benzene flux and (**b**) Separation factor (S.F.) at an initial benzene concentration of 550 ppm.

The response surface plot in Figure 10a illustrates the effect of the feed flow and the feed temperature on the benzene flux. The benzene permeate flux increased linearly with temperature as a result of increasing the flexibility. However, the flexible character of PDMS, due to its shortage of double bonds, allows a high degree of rotation of the bonds, facilitating the diffusion of permeating species through the free volume, leading to an increase in the membrane permeability with temperature [50]. Moreover, the benzene permeate flux increased slightly with the flow rate of the feed because this reduced the boundary layer over the membrane surface. The influence of the feed flow rate was evident after increasing the feed temperature from 30 to 50 °C.

The response surface plot in Figure 10b illustrates the effect of the feed flow and the feed temperature on the S.F. for the benzene–water mixture. As mentioned previously, due to membrane swelling, there was a negative effect of temperature on the S.F. Thus, when the temperature was raised the S.F. decreased. As a result, the amount of benzene permeation through the membrane was lower than that of water, while an increased feed flow rate increased the S.F. by reducing the boundary layer thickness of the surface membrane. Because the resistance of the boundary layer against the mass transfer on the upstream membrane decreased, the permeation flux of benzene rose.

**Figure 11:**
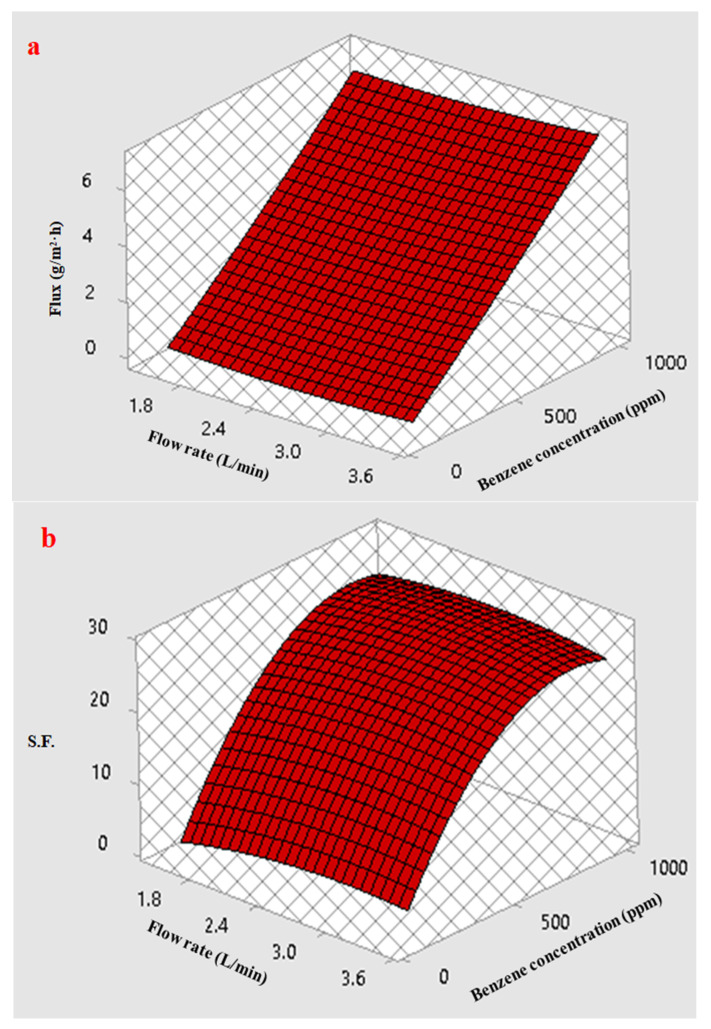
Effect of the feed flow and concentration on (**a**) benzene flux and (**b**) Separation factor (S.F.) at a temperature of 40 °C.

Figure 11a shows a three-dimensional display of the response surface plot for the benzene permeation flux with the coupling effect of the interaction between the feed concentration and the feed flow rate. Increasing the initial benzene concentration in the feed caused the benzene permeation flux to rise to a greater degree than did the feed flow rate. Generally, an increase in the benzene permeation flux results from the combined effect of an increase in the driving force (represented by the concentration difference across the membrane) and a weakening of the boundary layer adjacent to the membrane, where the total resistance against the permeation is composed of the boundary and membrane resistance. With an increase in the flow rate, the boundary layer thickness decreased, as did the boundary resistance.

The response surface plot in Figure 11b illustrates an effect of the initial concentration of benzene and the feed flow rate on the S.F. Increasing the benzene concentration in the feed increased the S.F. more than increasing the feed flow rate. This result may be explained by the fact that the water molecules clustered due to hydrogen bonding between water molecules, which reduced their diffusivity and permeability. However, increasing the initial concentration of benzene improved the S.F., even at a low feed flow rate.

## 4. Conclusions

Using PDMS membranes supplied by DeltaMem AG (Switzerland), soluble benzene was separated from water under various conditions of feed temperature, initial benzene concentration, and flow rate. This work evaluated the influences of these three factors on the permeate flux and separation factor. It was found that the flux increased with the temperature of the feed, the initial benzene concentration in the feed, and the feed flow rate, whereas the S.F. decreased with an increase in the feed temperature but rose with increases in the initial benzene concentration and feed flow rate. The separation of benzene compounds by the pervaporation process was studied using Minitab 18 and the RSM methodology. An analysis of variance and surface plots showed the combined effects of these factors on the permeate flux and separation factor and suggested a mathematical expression to calculate the flux and separation factor. Moreover, the optimized response was estimated based on composite desirability, where the feed temperature was 30 °C, the initial concentration of benzene was 1000 ppm, and the feed flow rate was 3.5 L/min. At these points, maximal output responses were both predicted and confirmed experimentally.

## Figures and Tables

**Table 1:** The performance of previous PV processes using PDMS membranes for the recovery of various VOCs from aqueous solutions.

Membrane	ThAL (µm)	ThSL (µm)	VOCs	T (°C)	Con.	Pressure	VOCs Flux (g/m^2^ h)	S.F.	Ref.
PDMS	87.5 ± 10.3	None	Ethanol	30	5 %wt.	@	11	10	[23]
PDMS_plasma_C_8_	126.5 ± 6.2	None					8	7	
(PDMS)-NaCl	167.5 ± 5.6	None					13	11	
PDMS_Al_2_O_3__nat	85.8 ± 11.2	None					10	10	
PDMS_Al_2_O_3__mod._C_8_	129.5 ± 10.9	None					21	11	
PDMS/PVDF	177.3 ± 13.2	24					8	9	
PDMS (Pervap^TM^4060)	5.5	178					201	9	
PDMS	@	@	Acetonitrile	40	5913 ppm	4–15 mbar	31.56	28.2	[25]
PDMS	250	None	Phenol	40	0.5 %wt.	200 Pa	4	15	[26]
				70			7	3	
PDMS + oleyl alcohol (5%)				40			3	3.5	
				70			11	6.5	
PDMS (Pervap^TM^4060)	5	169	Acetone	30	3 %wt.	5.5 kPa	2.7	100	[30]
				60			8.4	30	
			Acetonitrile	30			1.2	11	
				60			4.2	10	
			Ethanol	30			0.3	5	
				60			1.2	4	
PDMS + PES	20	200	Toluene	30	150 ppm	1 mbar	3.5	2200	[34]
				50	300 ppm		7.5	1300	
PDMS + PTFE	35	15	Acetone	30	0.99 %wt.	15 mmHg	8 (kg µm /m^2^ h)	55	[35]
			Butanone				10	125	
			Cyclohexane				6	85	
			Ethanol				6	5	
			Isopropanol				7	15	
			n-butyl alcohol				9	40	
			Acetic acid				6	2.5	
			Ethyl acetate				14	90	
PDMS + PVDF	32	25	Ethanol	35	9 %wt.	10 mmHg	367	6.6	[36]
PDMS	30	None	Butanol	55	1.5 %wt.		240	43	[37]

ThAL (thickness of active layer); ThSL (thickness of support layer); Con. (concentration); @ (unknown); S.F. (separation factor); PDMS (polydimethylsiloxane); PES (polyethersulfune); PVDF (polyvinylidene fluoride); PTFE (polytetrafluoroethylene).

**Table 2:** The performance of the present and previous PV processes for benzene recovery from an aqueous solution.

Membrane	ThAL (µm)	ThSL (µm)	T (°C)	Con.	Pressure	C_6_H_6_ Flux (g/m^2^ h)	S.F.	Ref.
PDMS	140–200	None	60	750 ppm	0.2 kPa	180	9000	[52]
PDMS	200	None	60	1400 ppm	1–10 kPa	126	3302	[53]
CA-f-PDMS	150					116	5604	
PDMS (composite)	100	100				365	4600	
CA-f-PDMS composite	50					407	5913	
PDMS + DMMA	270	None	40	500 ppm	0.01 mmHg	51.4	1853	[54]
PDMS + DVB	314					45.5	3099	
PDMS + DVS	276					70.9	2886	
PDMS + EGDM	357					49.6	2011	
PDMS + PES	11	None	25	150ppm	5 mbar	66	972	[55]
PDMS + PES	0.2	140	25	Benzene 2 %wt. + Methanol 50 %wt. in water	@	4.2	1	[56]
	0.5					7	1.5	
	2					6.4	2.5	
	3					7	4	
	8					7.3	7.5	
	35					8.1	15	
	150					10	20	
PDMS (Pervap^TM^4060)	5.5	178	30	1000 ppm	2 kPa	7.5	47	The present work

ThAL (thickness of active layer); ThSL (thickness of support layer); Con. (concentration); @ (unknown); S.F. (separation factor); PDMS (polydimethylsiloxane); PES (polyethersulfune); DMMA (dimethyl methacrylate macromonomer); DVB (divinylbenzene); DVS (divinylsiloxane); EGDM (ethylene glycol dimethyl methacrylate); CA (calixarene).

**Table 3:** Experimental data points and responses.

Std Order	Temp. (°C)	Conc. (ppm)	Flow Rate (L/min)	Flux (g/m^2^·h)	S.F.
6	50	100	3.5	0.24442	6.5231
12	40	1000	2.5	6.53818	23.7613
17	40	550	2.5	3.20917	23.7200
1	30	100	1.5	0.05733	2.2940
10	50	550	2.5	3.40000	18.8843
9	30	550	2.5	3.00000	32.3700
14	40	550	3.5	3.50000	20.0000
4	50	1000	1.5	7.05867	19.7256
13	40	550	1.5	3.12402	21.2793
3	30	1000	1.5	6.00000	36.9546
16	40	550	2.5	3.20917	23.7200
20	40	550	2.5	3.20917	23.7200
15	40	550	2.5	3.20917	23.7200
19	40	550	2.5	3.20917	23.7200
5	30	100	3.5	0.18333	8.8097
11	40	100	2.5	0.08583	4.1221
2	50	100	1.5	0.20035	6.2494
18	40	550	2.5	3.20917	23.7200
7	30	1000	3.5	6.77333	40.0000

**Table 4:** The coefficients in Equations (4) and (5).

	A_0_	A_1_	A_2_	A_3_	A_4_	A_5_	A_6_	A_7_	A_8_	A_9_
$J_{B}$	−0.662	0.0134	4.66 × 10^−3^	0.236	0.00125	1 × 10^−6^	0.1245	3.6 × 10^−5^	−0.00886	0.000208
S.F.	26	−2.467	0.11601	13.34	0.03609	−4 × 10^−5^	−1.378	−0.0011	−0.1247	−0.00123

**Table 5:** Analysis of variance for benzene permeate flux.

Source	DF	Adj SS	Adj MS	F-Value	*p*-Value
Model	9	108.871	12.097	2393.22	0.000
Linear	3	108.263	36.088	7139.59	0.000
Temp.	1	0.439	0.439	86.79	0.000
Conc.	1	107.610	107.610	21289.47	0.000
Flow	1	0.215	0.215	42.50	0.000
Square	3	0.268	0.089	17.65	0.000
Temp. × Temp.	1	0.000	0.000	0.09	0.776
Conc. × Conc.	1	0.043	0.043	8.44	0.016
Flow × Flow	1	0.043	0.043	8.44	0.016
2-Way Interaction	3	0.340	0.113	22.41	0.000
Temp. × Conc.	1	0.207	0.207	40.91	0.000
Temp. × Flow	1	0.063	0.063	12.43	0.005
Conc. × Flow	1	0.070	0.070	13.90	0.004
Error	10	0.051	0.005	-	-
Lack-of-Fit	5	0.051	0.010	-	-
Pure Error	5	0.000	0.000	-	-
Total	19	108.921	-	-	-

DF = Degrees of freedom, Adj SS = Sum of squares, Adj MS = Mean square, × means the multiplication sign.

**Table 6:** Analysis of variance for separation factor.

Source	DF	Adj SS	Adj MS	F-Value	*p*-Value
Model	9	1975.38	219.49	80.03	0.000
Linear	3	1499.05	499.68	182.19	0.000
Temp.	1	250.10	250.10	91.19	0.000
Conc.	1	1242.76	1242.76	453.12	0.000
Flow	1	6.19	6.19	2.26	0.164
Square	3	262.81	87.60	31.94	0.000
Temp. × Temp.	1	35.82	35.82	13.06	0.005
Conc. × Conc.	1	179.37	179.37	65.40	0.000
Flow × Flow	1	5.22	5.22	1.90	0.198
2-Way Interaction	3	213.52	71.17	25.95	0.000
Temp. × Conc.	1	198.62	198.62	72.42	0.000
Temp. × Flow	1	12.44	12.44	4.54	0.059
Conc. × Flow	1	2.46	2.46	0.90	0.366
Error	10	27.43	2.74	-	-
Lack-of-Fit	5	27.43	5.49	-	-
Pure Error	5	0.00	0.00	-	-
Total	19	2002.81	-	-	-

**Table 7:** Model summary for benzene permeate flux and separation factor.

Parameters	Stand. Dev.	R^2^	R^2^(adj)	R^2^(pred)
Permeate flux	0.0710957	99.95%	99.91%	99.11%
Separation factor	1.65611	98.63%	97.40%	90.47%

**Table 8:** Response optimization of benzene flux and S.F. for benzene–water solution.

Temp. (°C)	Conc. (ppm)	Flow (L/min.)	S.F.Fit	Flux Fit (g/m^2^·h)	Composite Desirability
30	1000	3.5	39.8049	6.70111	0.961608

## Data Availability

Not applicable.
